# Spatial Dissection of the Distinct Cellular Reponses to Normal Aging and Alzheimer’s Disease

**DOI:** 10.1002/alz.095387

**Published:** 2025-01-09

**Authors:** Yun Gong, Mohammad Haeri, Xiao Zhang, Yisu Li, Anqi Liu, Di Wu, Qilei Zhang, Xiaoxin Yan, S. Michal Jazwinski, Lindong Jiang, YiPing Chen, Russell H Swerdlow, Hui Shen, Hong‐Wen Deng

**Affiliations:** ^1^ Tulane University, New Orleans, LA USA; ^2^ University of Kansas Alzheimer’s Disease Research Center, Fairway, KS USA; ^3^ School of Basic Medical Sciences, Central South University, Changsha, Hunan China; ^4^ School of Basic Medical Science, Central South University, Changsha, Hunan China

## Abstract

**Background:**

Aging significantly elevates the risk for Alzheimer’s disease (AD), contributing to the accumulation of AD pathologies, such as amyloid‐β (Aβ), inflammation, and oxidative stress. The human prefrontal cortex (PFC) is highly vulnerable to the impacts of both aging and AD. Unveiling and understanding the molecular alterations in PFC associated with normal aging (NA) and AD is essential for elucidating the mechanisms of AD progression and developing novel therapeutics for this devastating disease.

**Method:**

In this study, for the first time, we employed a cutting‐edge spatial transcriptome platform, SpaTial Enhanced Resolution Omics‐sequencing (Stereo‐seq), to generate the first comprehensive, subcellular resolution spatial transcriptome atlas of the PFC from six AD cases at various neuropathological stages and six age, sex, and ethnicity matched controls.

**Result:**

Our analyses revealed distinct transcriptional alterations across six neocortex layers, highlighted the AD‐associated disruptions in laminar architecture, and identified changes in layer‐to‐layer interactions as AD progresses. Further, throughout the progression from NA to various stages of AD, we discovered specific genes that were significantly upregulated in neurons experiencing high stress and in nearby non‐neuronal cells, compared to cells distant from the source of stress. Notably, the cell‐cell interactions between the neurons under the high stress and adjacent glial cells that promote Aβ clearance and neuroprotection were diminished in AD in response to stressors compared to NA. Through cell‐type specific gene co‐expression analysis, we identified three modules in excitatory and inhibitory neurons associated with neuronal protection, protein dephosphorylation, and negative regulation of Aβ plaque formation. These modules negatively correlated with AD progression, indicating a reduced capacity for toxic substance clearance in AD subject samples. Moreover, we have discovered a novel transcription factor, ZNF460, that regulates all three modules, establishing it as a potential new therapeutic target for AD.

**Conclusion:**

Overall, utilizing the latest spatial transcriptome platform, our study developed the first transcriptome‐wide atlas with subcellular resolution for assessing the molecular alterations in the human PFC due to AD. This atlas sheds light on the potential mechanisms underlying the progression from NA to AD.

